# Risk factors and outcome in patients with primary sclerosing cholangitis with persistent biliary candidiasis

**DOI:** 10.1186/s12879-014-0562-8

**Published:** 2014-10-23

**Authors:** Christian Rupp, Konrad Alexander Bode, Fadi Chahoud, Andreas Wannhoff, Kilian Friedrich, Karl-Heinz Weiss, Peter Sauer, Wolfgang Stremmel, Daniel Nils Gotthardt

**Affiliations:** Department of Internal Medicine IV, University Hospital of Heidelberg, Im Neuenheimer Feld 410, Heidelberg, 69120 Germany; Department of Infectious Diseases, Medical Microbiology and Hygiene, University of Heidelberg, Im Neuenheimer Feld 324, Heidelberg, 69120 Germany

**Keywords:** Primary sclerosing cholangitis, Candida, Fungobilia, Biliary infection, Dominant stenosis, Liver transplantation, Cholestatic liver disease, Cholangiocarcinoma

## Abstract

**Background:**

Candidiasis is commonly observed in patients with primary sclerosing cholangitis (PSC), but the clinical risk factors associated with its presence have not been fully investigated. In this study, we aimed to analyse the incidence, risk factors, and transplantation-free survival in primary sclerosing cholangitis (PSC) patients with persistent biliary candidiasis.

**Methods:**

We retrospectively analysed patients diagnosed with PSC who were admitted to our department during 2002 to 2012. One-hundred fifty patients whose bile cultures were tested for fungal species were selected, and their clinical and laboratory parameters were investigated. The results of endoscopic retrograde cholangiography (ERC) and bile cultures were analysed using chart reviews. The cases of biliary candidiasis were sub-classified as transient or persistent.

**Results:**

Thirty out of 150 (20.0%) patients had biliary candidiasis. Although all patients demonstrated comparable baseline characteristics, those with biliary candidiasis showed significantly reduced transplantation-free survival (p < 0.0001) along with a markedly elevated frequency of cholangiocarcinoma (CCA) (p = 0.04). The patients were further sub-classified according to the transient (15/30) or persistent (15/30) nature of their biliary candidiasis. A subgroup analysis showed reduced survival with a greater necessity for orthotopic liver transplantation (OLT) only in patients with persistence of Candida (p = 0.007). The survival in the patients with transient biliary candidiasis was comparable to that in candidiasis-free patients. In a multivariate regression analysis that included Mayo risk score (MRS), sex, age, dominant stenosis, inflammatory bowel disease, autoimmune hepatitis overlap syndrome, and number of times ERC was performed, biliary candidiasis was an independent risk factor for reduced survival (p = 0.008). Risk factors associated with acquisition of biliary candidiasis were age at PSC diagnosis and number of ERCs.

**Conclusions:**

The persistence of biliary candidiasis is associated with markedly reduced transplantation-free survival in PSC patients. By contrast, actuarial survival in patients with transient biliary candidiasis approaches that for patients without any evidence of biliary candidiasis. Further studies on the treatment of persistent biliary candidiasis in patients with PSC are warranted.

**Electronic supplementary material:**

The online version of this article (doi:10.1186/s12879-014-0562-8) contains supplementary material, which is available to authorized users.

## Background

Primary sclerosing cholangitis (PSC) is a chronic cholestatic liver disease characterized by inflammation and stricture formation of the intra- and extrahepatic biliary system, which progresses to liver cirrhosis in the majority of cases [1]-[3]. There is a strong association with inflammatory bowel disease (IBD) in about 75% of patients, and a clear male predominance [4].

Therapeutic options for this condition are limited, and although the data regarding the survival benefits of ursodeoxycholic acid treatment are conflicting [5]-[8], it remains the most frequently used therapeutic agent. In end-stage disease, liver transplantation is the only curative therapy, and PSC remains a leading cause of liver transplantation, despite its low prevalence in Western countries [9]. The average time from first diagnosis to liver transplantation is about 12 to 15 years [10]. Unfortunately, the pathogenesis of PSC is still unresolved. Recent studies highlighted the influence of numerous genetic risk factors [11]-[13], and it is well accepted that immunological and environmental factors, such as transmission of bacterial pathogens from the gut due to increased permeability of the portal venous system, are also involved [14]-[16].

Owing to the limited medical treatment options, clinical management of PSC patients remains challenging, and identification of high-risk patients is an important problem for clinicians. Several clinical risk factors have been described that are associated with the reduced transplantation-free survival, e.g. dominant strictures and concomitant immunological diseases or biliary infections [17]-[19]. In addition to bacterial infections, some studies suggested a strong influence of fungal infections on the survival of PSC patients [20],[21]. Up to now, only *Candida* species have been described to cause fungal infections in PSC patients. It is well known that *Candida* species are frequently detected, especially in the presence of high-grade biliary strictures or tumour stenosis [22]. Treatment of fungal infection is difficult, and in many cases, eradication cannot be achieved [23].

In this study, we aimed to analyse clinical risk factors associated with the presence of biliary candidiasis in PSC patients. Furthermore, we sought to determine the influence of persistent candidiasis, compared with transient candidiasis, and to investigate its impact on survival.

## Methods

### Patients and study design

This study was designed to analyse clinical risk factors that are associated with biliary candidiasis and to assess how the outcome is influenced by the presence of transient and persistent biliary candidiasis. We screened all the PSC patients who were treated at the Heidelberg University Hospital between January 2002 and October 2012. During the study period, 290 PSC patients visited our department. The diagnosis of PSC was established on the basis of typical endoscopic retrograde cholangiography (ERC) findings, serum alkaline phosphatase activity of at least twice the reference range, negative antimitochondrial antibody, and results of liver biopsy compatible with the diagnosis of PSC. Only the patients with available results of endoscopic treatment and bile culture tests for fungal species were included in the final analysis. A total of 150 patients that satisfied the above selection criteria were followed until January 2013 (Table 1); Ninety-nine of the patients were diagnosed with PSC during their first ERC, performed in our department. Of the other 51 patients, 34 had dominant strictures treated by dilatation therapy before visiting our tertiary center. The frequency of strictures, numbers of bile cultures, and frequency of biliary candidiasis were not different between patients with their first ERC performed in our department and patients treated endoscopically prior to their first visit to our institution. 136 patients were excluded due to missing bile cultures tests for fungal infection. Four patients, diagnosed with CCA their first visit at our departement, were excluded because biliary candida was only detected after chemotherapy. The excluded patients showed a lower frequency of dominant stenosis and a trend for longer survival compared with our study cohort (log-rank, p = 0.1). The male/female ratio and the frequencies of CCA, liver transplantation, and death were not different between the study cohort and the excluded patients. Despite a lower level of alkaline phosphatase in our study cohort, compared with the excluded patients, there were no differences in baseline laboratory parameters, or in Mayo Risk Score, at baseline. The treatment of CCA involved liver transplantation for local disease (according to the Mayo-protocol) or partial liver resection. In advanced disease, chemotherapy was administered.

**Table 1:** **Baseline characteristics of the patients**

	N (%) or mean ± SD	Reference value
Patients	150	
Age at diagnosis (years)	32.0 ± 1.0	
Male sex	108 (72.0)	
IBD	108 (72.0)	
Overlap with autoimmune hepatitis	13 (8.7)	
Dominant stenosis	100 (66.7)	
Dominant stenosis at first diagnosis	22 (14.7)	
Number of ERCs	618	
Number of ERCs per patient	3.0 ± 0.3	
Laboratory parameters at baseline		
Bilirubin (mg/dL)	0.9 ± 0.3	-1.1
ALT (IU/L)	92.5 ± 9.6	-23
AST (IU/L)	63.7 ± 6.2	-19
AP (IU/L)	252.1 ± 20.2	-175
GGT (IU/L)	267.0 ± 28.5	-60
Albumin (g/dL)	42.0 ± 0.5	35-55
Mayo risk score	-0.12 ± 0.1	

IBD, inflammatory bowel disease; ALT, alanine aminotransferase; AST, aspartate aminotransferase; AP, alkaline phosphate; GGT, γ-glutamyltransferase; ERC, endoscopic retrograde cholangiography.

### Definitions

Transient candidiasis was defined as at least one bile culture positive for *Candida* sp., followed by at least one bile culture negative for biliary *Candida* and consecutive negative bile cultures until the end of the study. Persistent candidiasis was defined as bile cultures positive for *Candida* sp. until the end of study. Dominant stenosis was defined as a stenosis with a diameter smaller than 1.5-mm for the common duct or smaller than 1.0-mm in case of a hepatic duct (within 2 cm of the bifurcation). In patients with total or subtotal stenosis of the major duct, balloon dilatation of the stenosis was performed. Brush cytology was performed routinely in patients with an initial diagnosis of strictures during ERC and, furthermore, in any patient with suspected malignant disease. The end point was defined as liver transplantation or death.

### Antibiotic and antifungal treatment

For prevention of bacterial cholangitis as a consequence of ERC, the patients received peri-interventional antibiotic prophylaxis (mezlocillin or ciprofloxacin). The patients were not administered antibiotics before endoscopy, but received them intravenously directly after the aspiration of the bile sample and 1 week after the treatment. The patients with pre-existing bacterial cholangitis received intravenous mezlocillin, which was replaced with ciprofloxacin in patients with known allergy to penicillin, or further adapted according to an antibiogram.

Antifungal treatment was conducted for 8 out of 30 patients with *Candida* spp. present in the bile and who had clinical signs of cholangitis. Five patients were treated with fluconazole, two were treated with caspofungin, and one patient received voriconazole. Six out of 8 treated patients also had dominant stenosis. The patients without clinical signs of cholangitis and biliary candidiasis were not treated with specific antifungal agents because such a general recommendation did not exist at the time of the study.

### Bile cultures

All bile samples were obtained after selective bile duct intubation. Utilization of this method made contamination from the intestine unlikely. For all the patients, ERC was performed by an experienced gastroenterologist. Aliquots of all biliary samples were placed in sterile tubes and delivered to the microbiology laboratory within 2 hours. The samples were cultured aerobically at 37 °C for 48 hours on blood plates (Colombia agar with 5% sheep blood), chocolate agar, and CHROMagar™ Candida medium (all from Becton-Dickinson, Franklin Lakes, NJ, USA), as well as anaerobically using Schaedler agar (5% sheep blood, bioMerieux, Marcy l’Etoile, France). The cultures were incubated for 48 hours, with the first reading taken after 24 hours.

### Statistical analysis

Calculations were carried out using PASW Statistics 20. Frequencies were compared using a chi-square test. Continuous data were compared using the nonparametric Wilcoxon rank-sum test or the Fisher’s exact test, where appropriate. Actuarial transplantation-free survival was estimated using a Kaplan-Meier product limit estimator. Differences between the actuarial estimates were tested using the log rank test. Factors that independently affected the risk of reduced transplantation-free survival were determined using Cox proportional hazard ratio models with simultaneous adjustment for Mayo risk score, sex, age at first diagnosis, presence of IBD, presence of dominant stenosis, concomitant autoimmune hepatitis overlap syndrome, number of ERCs, and biliary candidiasis. Furthermore, we used Cox proportional hazard ratio models to explore the relative risks of biliary candidiasis with regard to clinical risk factors such as sex, age at diagnosis of PSC, presence of dominant stenosis, presence of IBD, Mayo risk score, and number of ERCs; p-values < 0.05 were considered statistically significant.

### Informed consent

Written informed consent was obtained from each patient, and the study protocol conformed to the ethical guidelines of the Declaration of Helsinki in its current version, as reflected in a priori approval by the institution’s human research review committee. The study was approved by the local ethics committee of Heidelberg University.

## Results

### Clinical and laboratory characteristics at baseline

**Figure 1:**
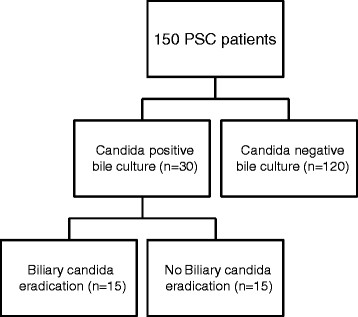
**Schematic representation of the study design.**

A cohort of 150 patients with PSC was analysed in this study. One hundred eight (72.0%) patients were men and 42 (28.0%) were women. One hundred eight (72.0%) patients had concomitant IBD and 100 (66.7%) showed dominant stenosis. Of the patients with dominant stenosis, 22 (14.7%) displayed this during their initial PSC diagnosis and 78 (52.0%) developed dominant stenosis during the course of their disease. The median age of diagnosis was 32.0 years. Other clinical and laboratory baseline characteristics are summarized in Table 1. Bile cultures were routinely collected from all the patients during ERC and tested for the presence of bacterial and fungal species. On average 3.0 ± 0.3 (median ± SEM) bile cultures were collected for each patient. Bacterial strains were identified in bile cultures of 105 (70.0%) patients. involving Enterococcus sp., in the majority of cases. One hundred twenty (80.0%) patients had no evidence of biliary candidiasis throughout the course of the study, whereas 30 (20.0%) patients had at least one bile culture positive for *Candida* spp.; *C. albicans* was detected in 25 patients, *C. glabrata* was identified in three patients; both *C. kruzei* and *C. spherica* were detected in one patient, and both *C.kruzei* and *C. albicans* were found in one patient. In five of those patients, only *Candida* sp. were identified during the course of disease. Six patients had candida identified in their initial bile culture. All of these patients had their first ERC in our department. In five other patients, the candida were biliary pathogens that were first identified after a prior, sterile bile culture. Four additional patients did not have antibiotic treatment prior to detection of biliary candidiasis. The remaining fifteen patients had endoscopic treatment and antibiotic therapy due to dominant stenosis and episodes of cholangitis. None of the patients included in the study received long-term antibiotic therapy. The patients were further sub-classified according to the transient (n = 15) or persistent (n = 15) nature of candidiasis (Figure 1).

### Clinical characteristics and clinical course with regard to the presence of biliary candidiasis

Although there were no significant differences in the baseline clinical and laboratory parameters between the patients with *Candida*-positive and *Candida*-negative bile cultures, except for elevated levels of γ-glutamyltransferase (p = 0.05) at inclusion into the study, such differences were found when the sub-division of candidiasis into transient and persistent forms was taken into account. Only the patients with the transient form showed elevated levels of γ-glutamyltransferase compared to that of the patients without candidiasis (p = 0.01). Furthermore, the patients with persistent candidiasis had higher albumin levels at baseline than the individuals without candidiasis (p = 0.04). The other clinical and laboratory parameters did not vary between the subgroups. We further analysed the clinical course of the patients with regard to the presence and persistence of biliary *Candida*. Although the frequency of dominant stenosis was similar between the subgroups, the patients with persistent candidiasis developed re-stenosis of the large bile ducts significantly more often than the patients with transient candidiasis (14/15 vs. 9/15; p = 0.031). The frequency of dominant stenosis at the initial PSC diagnosis and at subsequent time points were not different between the subgroups (Table 2). Eight patients received specific antifungal treatment, including five treated with fluconazole, two with caspofungin, and one with voriconazole. Of those patients, one fluconazole-treated patient, both caspofungin-treated patients, and the patient receiving voriconazole achieved clearance of the candida from their bile fluid. Candida treatment was not associated with eradication of biliary candidiasis (4/15 vs. 4/15, p = 1.0) or improved transplantation-free survival (mean: 14.3 ± 1.5 years vs. 13.8 ± 4.3 years; p = 0.9) (Table 3).

**Table 2:** **Clinical characteristics with regard to the presence of Candida in the bile fluid**

	No Candida	Candida	Transient Candida	Persistent Candida	Reference value
Patients	120	30	15	15	
Age at diagnosis (years)	32.0 ± 1.1*	31.0 ± 2.3	37.0 ± 3.6	27.0 ± 2.9	
Male sex	87 (72.5)	21 (70.0)	9 (60.0)	12 (80.0)	
IBD	86 (71.7)	22 (73.3)	10 (66.7)	12 (80.0)	
Overlap with autoimmune hepatitis	11 (9.2)	2 (6.7)	2 (13.3)	0 (0.0)	
Dominant stenosis	76 (63.3)	24 (80.0)	11 (86.7)	13 (73.3)	
- at first diagnosis	17 (14.2)	5 (16.7)	2 (13.3)	3 (20.0)	
- time from first diagnosis, years	5.9 ± 5.7	5.6 ± 4.5	5.6 ± 4.9	5.6 ± 4.4	
Number of ERCs per patient	3.0 ± 0.3	4.0 ± 0.6	4.0 ± 0.7	4.0 ± 1.0	
Laboratory parameters at baseline					
Bilirubin (mg/dL)	0.9 ± 0.3	0.9 ± 0.9	1.0 ± 0.7	0.8 ± 1.6	-1.1
ALT (IU/L)	88.7 ± 10.5	118.0 ± 23.2	104.6 ± 45.6	124 ± 12.8	-23
AST (IU/L)	53.9 ± 6.2	82.0 ± 18.3	90.4 ± 24.4	70.0 ± 28.1	-19
AP (IU/L)	240.4 ± 23.0	266.5 ± 41.6	270.0 ± 80.0	265.0 ± 24.3	-175
GGT (IU/L)	247.5 ± 31.1	344.5 ± 67.7^a^	448.0 ± 112.9^a^	279 ± 62.6	-60
Albumin (g/dL)	41.0 ± 0.6	45.0 ± 1.5	41.0 ± 2.7	45.0 ± 1.3^a^	35-55
Mayo Risk Score	-0.25 ± 0.1	-0.28 ± 0.3	0.04 ± 0.3	-0.3 ± 0.4	
Clinical outcome					
Development of CCA	6 (5.0)	5 (16.7)^a^	4 (26.7)^a^	1 (6.7)	
Death	8 (6.7)	4 (13.3)	3 (20.0)	1 (6.7)	
OLT	20 (16.8)	14 (46.7)^a^	5 (33.3)	9 (60.0)^a^	
Death/OLT	28 (23.3)	18 (60.0)^a^	8 (53.3)	10 (66.7)^a^	
Survival free of liver transplantation (years)	20.1 ± 2.1	11.5 ± 2.6^a^	17.8 ± 7.3	11.1 ± 2.1^a^	

IBD, inflammatory bowel disease; ALT, alanine aminotransferase; AST, aspartate aminotransferase; AP, alkaline phosphate; GGT, γ-glutamyltransferase; ERC, endoscopic retrograde cholangiography; OLT, orthotopic liver transplantation; CCA, cholangiocarcinoma.

*Data are presented as N (%) or mean ± SD.

^a^Significantly different from patients without candida (p <0.05).

**Table 3:** **Characteristics associated with identification of specific fungal species; all values represent N**

	*C. albicans*	*C. glabrata*	*C. kruzei*	*C. tropicalis*	*C. spherica*
Patients	25	3	2*	1	1
*Candida* only	4	0	1*	1	0
Additional bacteria in bile	21	3	1	0	1
Dominant stenosis	20	2	2	1	1
Dominant stenosis at first diagnosis	5	0	0	0	0
Cholangiocarcinoma	5	0	1*	0	1
Anti-fungal treatment	7	1	0	0	0
Clearance of candidiasis	3	1	0	0	0

*In one patient *C. kruzei* and *C. tropicalis* were concomitantly detected. In another patient *C. kruzei* and *C. spherica* were concomitantly detected.

### Outcome analysis

**Figure 2:**
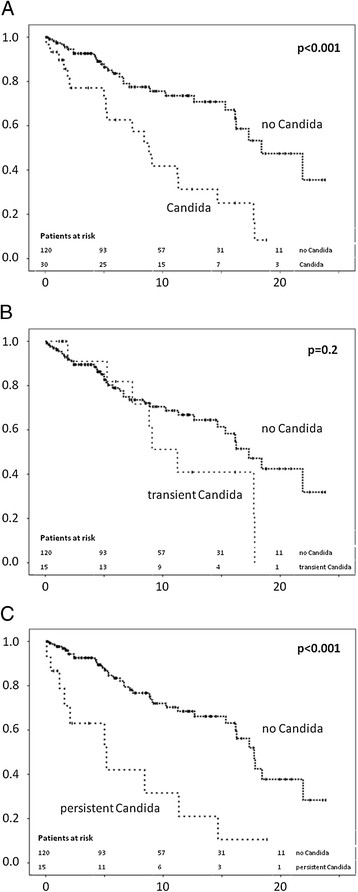
**Kaplan-Meier analysis of actuarial transplantation-free survival (n = 150).** The survival is given in years from the time of first diagnosis. Patients with biliary candidiasis had reduced survival compared to patients without candidiasis **(A)**. Patients with transient biliary candidiasis showed no difference in survival rates **(B)**, whereas patients with persistent biliary candidiasis had markedly reduced survival **(C)**.

The patients with bile cultures positive for *Candida* showed a markedly elevated frequency of CCA (p = 0.04), although there was no difference between the 2 subgroups. The mean time interval between detection of *Candida* in the bile and CCA diagnosis was 2.2 years. Of note, three of the five patients with CCA were diagnosed with candidiasis at the same time as their initial CCA diagnosis. Patients with candidiasis needed orthotopic liver transplantation (OLT) more often, and they more often reached the combined endpoint (OLT: p = 0.007; death/OLT combined: p < 0.0001). All patients died due to CCA. A subgroup analysis revealed elevated frequencies of OLT and reaching the combined endpoint only in the patients with persistent candidiasis (p = 0.003 and p = 0.004, respectively) (Table 2). These results were confirmed by Kaplan-Meier analysis. The patients with candidiasis showed a markedly reduced transplantation-free survival than those without *Candida* (log-rank: p < 0.001). Remarkably, only the patients with persistent candidiasis showed reduced transplantation-free survival (log-rank: p = 0.002) (Figure 2A-C).

### Assessment of risk factors for reduced transplantation-free survival and biliary candidiasis

In order to identify clinical risk factors for acquisition of biliary candidiasis, we performed univariate and multivariate Cox regression analyses. We included the following potential risk factors: sex, presence of dominant stenosis, presence of IBD, autoimmune hepatitis overlap syndrome, age at diagnosis of PSC, number of ERCs, and Mayo risk score at baseline. In the univariate regression analysis, the presence of dominant stenosis (p = 0.05), age at diagnosis (p = 0.02), and number of ERCs (p = 0.002) were associated with biliary candidiasis. In the multivariate analysis, only age at diagnosis (p = 0.019) and number of ERCs (p = 0.025) were independently associated with biliary candidiasis (Table 4).

**Table 4:** **Risk factors of biliary candidiasis**

Risk factor	Univariate			Multivariate		
	HR	HR (95% CI)	p-value	HR	HR (95% CI)	p-value
Sex	1.8	0.8-4.0	0.2	1.5	0.5-4.1	0.5
Presence of dominant stenosis	0.3	0.1-1.0	**0.05**	0.6	0.1-2.6	0.4
Presence of IBD	1.1	0.5-2.4	0.9	0.7	0.3-2.1	0.5
Overlap with autoimmune hepatitis	1.0	0.2-4.1	0.9	0.6	0.1-6.8	0.7
Age at PSC diagnosis	1.0	1.0-1.1	**0.021**	1.0	1.0-1.1	**0.019**
Number of ERCs	1.2	1.1-1.4	**0.002**	1.2	1.0-1.4	**0.025**
Mayo Risk Score	0.9	0.7-1.3	0.7	0.9	0.6-1.3	0.6

HR, hazard ratio; CI, confidence interval; ERC, endoscopic retrograde cholangiography; IBD, inflammatory bowel disease; PSC, primary sclerosing cholangitis; AIHOLS, autoimmune hepatitis overlap syndrome; DS, dominant stenosis.

The data show prospective factors predicting the development of biliary candidiasis. In the univariate analysis, the presence of dominant stenosis, number of ERCs, and age at diagnosis of PSC were associated with biliary candidiasis. In the multivariate analysis, only the number of ERCs and age at diagnosis of PSC reached significance (p < 0.05, bold).

Finally, we evaluated the influence of the same clinical risk factors together with the presence of biliary candidiasis on actuarial transplantation-free survival in our study cohort. In the univariate analysis, Mayo risk score (p = 0.02) and biliary candidiasis (p = 0.006) were associated with reduced survival, whereas presence of dominant stenosis and IBD reached borderline significance. In the multivariate analysis, presence of dominant stenosis (p =0.04), presence of IBD (p = 0.02), Mayo risk score (p = 0.01), and biliary candidiasis (p = 0.008) were independently associated with reduced survival (Table 5).

**Table 5:** **Risk factors of reduced transplantation-free survival**

Risk factor	Univariate			Multivariate		
	HR	HR (CI 95%)	p-value	HR	HR (CI 95%)	p-value
Sex	0.8	0.4-1.6	0.5	1.2	0.5-2.6	0.7
Presence of dominant stenosis	0.5	0.2-1.0	0.06	0.4	0.1-1.0	**0.04**
Presence of IBD	0.5	0.2-1.1	0.07	0.3	0.1-0.8	**0.02**
Overlap with autoimmune hepatitis	0.9	0.3-2.8	0.8	0.4	0.1-1.5	0.2
Age at PSC diagnosis	1.0	1.0-1.1	0.3	1.0	1.0--1.1	0.09
Number of ERCs	1.0	0.9-1.1	0.8	1.0	0.9-1.1	0.7
Mayo Risk Score	1.3	1.1-1.7	**0.02**	1.4	1.1-1.8	**0.01**
Candida	0.4	0.2-0.8	**0.006**	0.4	0.2-0.8	**0.008**

HR, hazard ratio; CI, confidence interval; ERC, endoscopic retrograde cholangiography; IBD, inflammatory bowel disease; PSC, primary sclerosing cholangitis; AIHOLS, autoimmune hepatitis overlap syndrome; DS, dominant stenosis.

The data show prospective factors predictive of longer actuarial transplantation-free survival. In the univariate analysis, the presence of dominant stenosis, number of ERCs, and age at diagnosis of PSC were associated with biliary candidiasis. In the multivariate analysis, only the number of ERCs and age at diagnosis of PSC reached significance (p < 0.05, bold).

## Discussion

In this large PSC cohort, we were able to identify clinical risk factors associated with acquisition of biliary candidiasis. Furthermore, we validated the negative influence of biliary candidiasis in PSC patients. This effect was especially emphasised in the patients with persistence of biliary candida throughout the course of the disease, whereas eradication of candida from the bile fluid resulted in survival rates almost comparable to those of patients without biliary candidiasis. Successful dilatation of biliary obstructions may contribute to candida eradication.

Several studies demonstrated that pathogenic antigens infiltrating the liver are capable of inducing hepatobiliary inflammation with numerous features of PSC. In particular, the increased enterohepatic circulation of pathogenic antigens as a consequence of abnormal intestinal permeability can contribute to the pathogenesis of PSC [15],[24],[25]. In this regard, portal bacteremia and bacterobilia were demonstrated in PSC patients with concomitant IBD [26]. Several studies of antibiotic treatment of PSC provided evidence for a beneficial effect on biochemical parameters and the resolution of symptoms [27]. Chronic alterations in the composition of the microbiome were shown to contribute to the pathogenesis of IBD and may also play a pivotal role in PSC [28].

In addition to bacterial infections common for PSC, fungal infections are becoming an increasingly recognized complication associated with a poor prognosis. The frequency of fungal pathogens in the bile fluid ranges from 15 to 50%, depending on the underlying disease [19],[22],[29]. The persistence of candida has already been associated with a worse prognosis in neonates [30]. In this study, we were able to detect *Candida* sp. in 20% of all patients. Furthermore, these patients showed markedly reduced transplantation-free survival when compared to the patients without candidiasis. Similar results have been described before, especially in immunosuppressed patients, after liver transplantation or with haematological malignancies [31]-[33]. Importantly, in the eight patients undergoing concomitant blood and bile cultures, *Candida* spp. were not detected in any blood cultures; underscoring the importance of standardized bile culture sampling [34]. In our study cohort, *C. albicans* was the most frequent fungal species isolated, detectable in 25 of 30 patients. If non-albicans species were excluded from the survival analysis, the results become even more significant, indicating a pathogenetic role for *C. albicans*. Of note, the negative effect of candida on survival in our cohort was independent of the number of interventions, although the number of interventions was a risk factor for the acquisition of biliary fungal infections. The negative effect of candidiasis was also independent of dominant stenosis, indicating that the pathological effect of candidiasis is likely independent of biliary obstructions caused by dominant stenosis. In line with this finding, dilatation therapy for dominant stenosis was more successful in patients with the transient form of candidiasis, and there was less need to repeat the endoscopic therapy. Therefore, the persistence of *Candida* species may be, in part, explained by the impaired biliary clearance in patients with persistent biliary candidiasis [35], but may also be an indication of compromised host defence [33],[36],[37]. Importantly, this condition seems to be resolved by liver transplantation, as only two out of fourteen patients had evidence of candida in their bile fluid after liver transplantation.

Clinical risk factors associated with the acquisition of biliary fungal infections are important for reasonable and cost effective screening strategies. Our analysis identified repeated therapeutic endoscopic interventions as well as older age at the time of diagnosis as independent risk factors associated with biliary candidiasis. Importantly, diagnostic ERCs do not confer an increased risk of biliary fungal infections in our cohort, as also been shown by other groups [22].

The interpretation of our results is limited by the retrospective nature of the study and the lack of a consistent antifungal treatment protocol. Furthermore, there might be a possibility of bias, as patients with strictures received more endoscopic interventions and subsequent bile samples compared with patients without dominant strictures. Nevertheless, since all the patients showed comparable baseline characteristics, and the frequencies of the endoscopic dilatation therapy and antifungal therapy were equal between the subgroups, we believe that the main conclusions of this study are reliable, and antifungal therapy should be considered for all patients with biliary candidiasis.

## Conclusion

In conclusion, we identified persistent biliary candidiasis as an independent risk factor of reduced transplantation-free survival in PSC patients. Clearance of biliary *Candida* results in improved survival, which is comparable to that of patients without fungobilia. Therefore, we recommend performing routine bile aspirations during ERC, followed by microbial analyses, including screening for fungal species. An aggressive antifungal treatment should be conducted if fungal pathogens are identified in these patients. Larger, prospectively designed studies are needed to confirm the beneficial effect of antifungal treatment in PSC patients.

## Authors’ original submitted files for images

Below are the links to the authors’ original submitted files for images. Authors’ original file for figure 1 Authors’ original file for figure 2 Authors’ original file for figure 3 Authors’ original file for figure 4

## Abbreviations

PCS: Primary sclerosing cholangitis
CCA: Cholangiocarcinoma
ERC: Endoscopic retrograde cholangiography
IBD: Inflammatory bowel disease
OLT: Orthotopic liver transplantation

## References

[1] Hirschfield GM, Karlsen TH, Lindor KD, Adams DH (2013). Primary sclerosing cholangitis. Lancet.

[2] Chapman R, Fevery J, Kalloo A, Nagorney DM, Boberg KM, Shneider B, Gores GJ (2010). Diagnosis and management of primary sclerosing cholangitis. Hepatology.

[3] Eaton JE, Talwalkar JA, Lazaridis KN, Gores GJ, Lindor KD (2013). Pathogenesis of primary sclerosing cholangitis and advances in diagnosis and management. Gastroenterology.

[4] Karlsen TH, Boberg KM (2013). Update on primary sclerosing cholangitis. J Hepatol.

[5] Imam MH, Sinakos E, Gossard AA, Kowdley KV, Luketic VA, Edwyn Harrison M, McCashland T, Befeler AS, Harnois D, Jorgensen R, Petz J, Keach J, DeCook AC, Enders F, Lindor KD (2011). High-dose ursodeoxycholic acid increases risk of adverse outcomes in patients with early stage primary sclerosing cholangitis. Aliment Pharmacol Ther.

[6] Lindor KD, Kowdley KV, Luketic VA, Harrison ME, McCashland T, Befeler AS, Harnois D, Jorgensen R, Petz J, Keach J, Mooney J, Sargeant C, Braaten J, Bernard T, King D, Miceli E, Schmoll J, Hoskin T, Thapa P, Enders F (2009). High-dose ursodeoxycholic acid for the treatment of primary sclerosing cholangitis. Hepatology.

[7] Pardi DS, Loftus EV, Kremers WK, Keach J, Lindor KD (2003). Ursodeoxycholic acid as a chemopreventive agent in patients with ulcerative colitis and primary sclerosing cholangitis. Gastroenterology.

[8] Beuers U, Spengler U, Kruis W, Aydemir U, Wiebecke B, Heldwein W, Weinzierl M, Pape GR, Sauerbruch T, Paumgartner G (1992). Ursodeoxycholic acid for treatment of primary sclerosing cholangitis: a placebo-controlled trial. Hepatology.

[9] Singal AK, Guturu P, Hmoud B, Kuo YF, Salameh H, Wiesner RH (2013). Evolving frequency and outcomes of liver transplantation based on etiology of liver disease. Transplantation.

[10] Boonstra K, Weersma RK, van Erpecum KJ, Rauws EA, Spanier BW, Poen AC, van Nieuwkerk KM, Drenth JP, Witteman BJ, Tuynman HA, Naber AH, Kingma PJ, van Buuren HR, van Hoek B, Vleggaar FP, van Geloven N, Beuers U, Ponsioen CY (2013). Population-based epidemiology, malignancy risk, and outcome of primary sclerosing cholangitis. Hepatology.

[11] Karlsen TH, Franke A, Melum E, Kaser A, Hov JR, Balschun T, Lie BA, Bergquist A, Schramm C, Weismuller TJ, Gotthardt D, Rust C, Philipp EE, Fritz T, Henckaerts L, Weersma RK, Stokkers P, Ponsioen CY, Wijmenga C, Sterneck M, Nothnagel M, Hampe J, Teufel A, Runz H, Rosenstiel P, Stiehl A, Vermeire S, Beuers U, Manns MP, Schrumpf E (2010). Genome-wide association analysis in primary sclerosing cholangitis. Gastroenterology.

[12] Ellinghaus D, Folseraas T, Holm K, Ellinghaus E, Melum E, Balschun T, Laerdahl JK, Shiryaev A, Gotthardt DN, Weismuller TJ, Schramm C, Wittig M, Bergquist A, Bjornsson E, Marschall HU, Vatn M, Teufel A, Rust C, Gieger C, Wichmann HE, Runz H, Sterneck M, Rupp C, Braun F, Weersma RK, Wijmenga C, Ponsioen CY, Mathew CG, Rutgeerts P, Vermeire S (2013). Genome-wide association analysis in primary sclerosing cholangitis and ulcerative colitis identifies risk loci at GPR35 and TCF4. Hepatology.

[13] Liu JZ, Hov JR, Folseraas T, Ellinghaus E, Rushbrook SM, Doncheva NT, Andreassen OA, Weersma RK, Weismuller TJ, Eksteen B, Invernizzi P, Hirschfield GM, Gotthardt DN, Pares A, Ellinghaus D, Shah T, Juran BD, Milkiewicz P, Rust C, Schramm C, Muller T, Srivastava B, Dalekos G, Nothen MM, Herms S, Winkelmann J, Mitrovic M, Braun F, Ponsioen CY, Croucher PJ (2013). Dense genotyping of immune-related disease regions identifies nine new risk loci for primary sclerosing cholangitis. Nat Genet.

[14] Andersen IM, Tengesdal G, Lie BA, Boberg KM, Karlsen TH, Hov JR (2014). Effects of Coffee Consumption, Smoking, and Hormones on Risk for Primary Sclerosing Cholangitis. Clin Gastroenterol Hepatol.

[15] Katt J, Schwinge D, Schoknecht T, Quaas A, Sobottka I, Burandt E, Becker C, Neurath MF, Lohse AW, Herkel J, Schramm C (2013). Increased T helper type 17 response to pathogen stimulation in patients with primary sclerosing cholangitis. Hepatology.

[16] Chapman R, Cullen S (2008). Etiopathogenesis of primary sclerosing cholangitis. World J Gastroenterol.

[17] Gotthardt DN, Rudolph G, Kloters-Plachky P, Kulaksiz H, Stiehl A (2010). Endoscopic dilation of dominant stenoses in primary sclerosing cholangitis: outcome after long-term treatment. Gastrointest Endosc.

[18] Rupp C, Mummelthei A, Sauer P, Weiss KH, Schirmacher P, Stiehl A, Stremmel W, Gotthardt DN (2013). Non-IBD immunological diseases are a risk factor for reduced survival in PSC. Liver Int.

[19] Negm AA, Schott A, Vonberg RP, Weismueller TJ, Schneider AS, Kubicka S, Strassburg CP, Manns MP, Suerbaum S, Wedemeyer J, Lankisch TO (2010). Routine bile collection for microbiological analysis during cholangiography and its impact on the management of cholangitis. Gastrointest Endosc.

[20] Rudolph G, Gotthardt D, Kloters-Plachky P, Kulaksiz H, Rost D, Stiehl A (2009). Influence of dominant bile duct stenoses and biliary infections on outcome in primary sclerosing cholangitis. J Hepatol.

[21] Pohl J, Ring A, Stremmel W, Stiehl A (2006). The role of dominant stenoses in bacterial infections of bile ducts in primary sclerosing cholangitis. Eur J Gastroenterol Hepatol.

[22] Lenz P, Conrad B, Kucharzik T, Hilker E, Fegeler W, Ullerich H, Heinecke A, Domschke W, Domagk D (2009). Prevalence, associations, and trends of biliary-tract candidiasis: a prospective observational study. Gastrointest Endosc.

[23] Domagk D, Fegeler W, Conrad B, Menzel J, Domschke W, Kucharzik T (2006). Biliary tract candidiasis: diagnostic and therapeutic approaches in a case series. Am J Gastroenterol.

[24] Bjornsson E, Cederborg A, Akvist A, Simren M, Stotzer PO, Bjarnason I (2005). Intestinal permeability and bacterial growth of the small bowel in patients with primary sclerosing cholangitis. Scand J Gastroenterol.

[25] Barrie A, Mourabet ME, Weyant K, Clarke K, Gajendran M, Rivers C, Park SY, Hartman D, Saul M, Regueiro M, Yadav D, Binion DG (2013). Recurrent blood eosinophilia in ulcerative colitis is associated with severe disease and primary sclerosing cholangitis. Dig Dis Sci.

[26] O’Toole A, Alakkari A, Keegan D, Doherty G, Mulcahy H, O’Donoghue D (2012). Primary sclerosing cholangitis and disease distribution in inflammatory bowel disease. Clin Gastroenterol Hepatol.

[27] Tabibian JH, Weeding E, Jorgensen RA, Petz JL, Keach JC, Talwalkar JA, Lindor KD (2013). Randomised clinical trial: vancomycin or metronidazole in patients with primary sclerosing cholangitis - a pilot study. Aliment Pharmacol Ther.

[28] Tabibian JH, Talwalkar JA, Lindor KD (2013). Role of the microbiota and antibiotics in primary sclerosing cholangitis. Biomed Res Int.

[29] Rupp C, Friedrich K, Folseraas T, Wannhoff A, Bode KA, Weiss KH, Schirmacher P, Sauer P, Stremmel W, Gotthardt DN (2014). Fut2 genotype is a risk factor for dominant stenosis and biliary candida infections in primary sclerosing cholangitis. Aliment Pharmacol Ther.

[30] Hammoud MS, Al-Taiar A, Fouad M, Raina A, Khan Z (2013). Persistent candidemia in neonatal care units: risk factors and clinical significance. Int J Infect Dis.

[31] Gotthardt DN, Weiss KH, Rupp C, Bode K, Eckerle I, Rudolph G, Bergemann J, Kloeters-Plachky P, Chahoud F, Buchler MW, Schemmer P, Stremmel W, Sauer P (2013). Bacteriobilia and fungibilia are associated with outcome in patients with endoscopic treatment of biliary complications after liver transplantation. Endoscopy.

[32] Wiesner RH, Hermans PE, Rakela J, Washington JA, Perkins JD, DiCecco S, Krom R (1988). Selective bowel decontamination to decrease gram-negative aerobic bacterial and Candida colonization and prevent infection after orthotopic liver transplantation. Transplantation.

[33] Zirkel J, Klinker H, Kuhn A, Abele-Horn M, Tappe D, Turnwald D, Einsele H, Heinz WJ (2012). Epidemiology of Candida blood stream infections in patients with hematological malignancies or solid tumors. Med Mycol.

[34] Nunes CZ, Marra AR, Edmond MB, da Silva VE, Pereira CA (2013). Time to blood culture positivity as a predictor of clinical outcome in patients with Candida albicans bloodstream infection. BMC Infect Dis.

[35] Katz S, Merkel GJ, Folkening WJ, Rosenthal RS, Grosfeld JL (1991). Impaired clearance and organ localization of Candida albicans in obstructive jaundice. J Pediatr Surg.

[36] Fei M, Bhatia S, Oriss TB, Yarlagadda M, Khare A, Akira S, Saijo S, Iwakura Y, Fallert Junecko BA, Reinhart TA, Foreman O, Ray P, Kolls J, Ray A (2011). TNF-alpha from inflammatory dendritic cells (DCs) regulates lung IL-17A/IL-5 levels and neutrophilia versus eosinophilia during persistent fungal infection. Proc Natl Acad Sci U S A.

[37] Smeekens SP, Ng A, Kumar V, Johnson MD, Plantinga TS, van Diemen C, Arts P, Verwiel ET, Gresnigt MS, Fransen K, van Sommeren S, Oosting M, Cheng SC, Joosten LA, Hoischen A, Kullberg BJ, Scott WK, Perfect JR, van der Meer JW, Wijmenga C, Netea MG, Xavier RJ (2013). Functional genomics identifies type I interferon pathway as central for host defense against Candida albicans. Nat Commun.

