# Supplementary figures and images for: Risk factors and outcome in patients with primary sclerosing cholangitis with persistent biliary candidiasis
Source: BMC Infect Dis. 2014 Oct 23;14:562. doi: 10.1186/s12879-014-0562-8 (PMC4209225; doi:10.1186/s12879-014-0562-8)

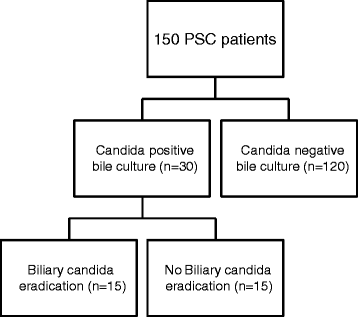

Supplement: Supplementary file 1 — Authors’ original file for figure 1 [file 12879_2014_562_MOESM1_ESM.gif]

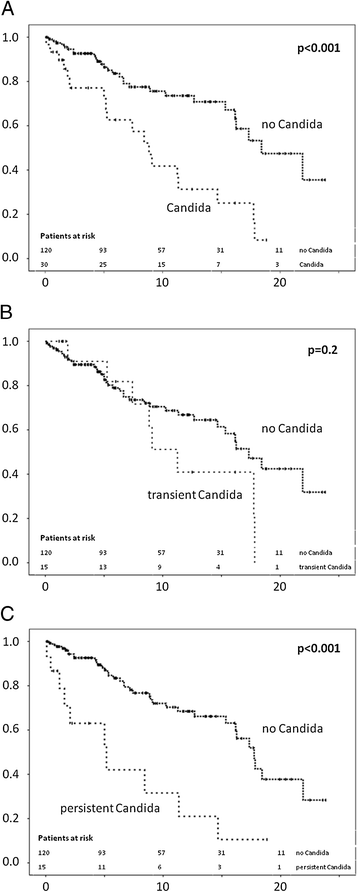

Supplement: Supplementary file 2 — Authors’ original file for figure 2 [file 12879_2014_562_MOESM2_ESM.gif]

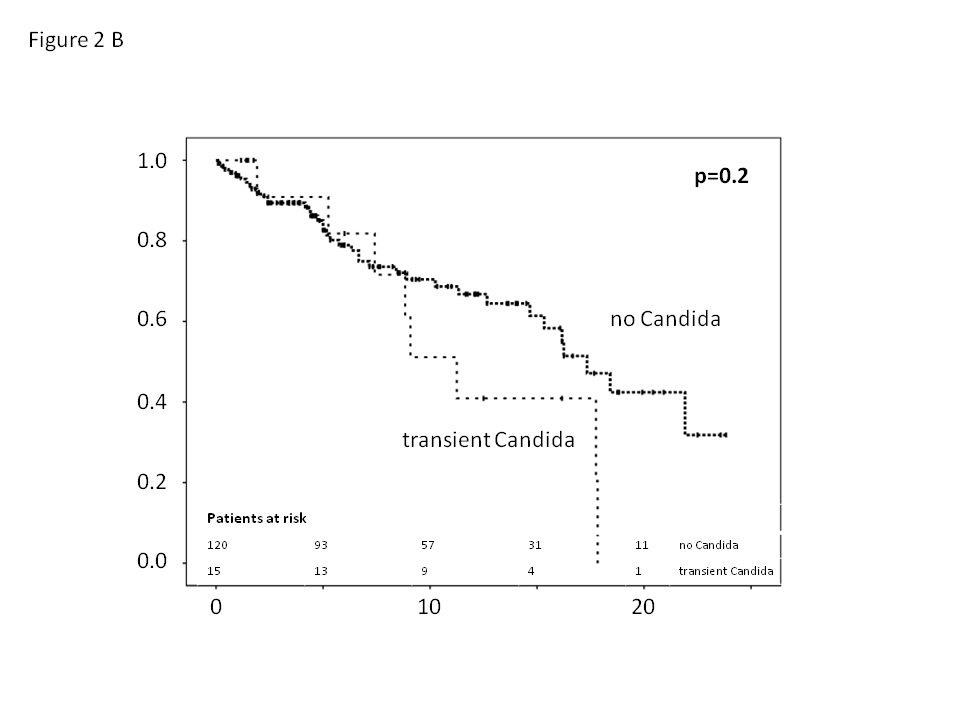

Supplement: Supplementary file 3 — Authors’ original file for figure 3 [file 12879_2014_562_MOESM3_ESM.tiff]

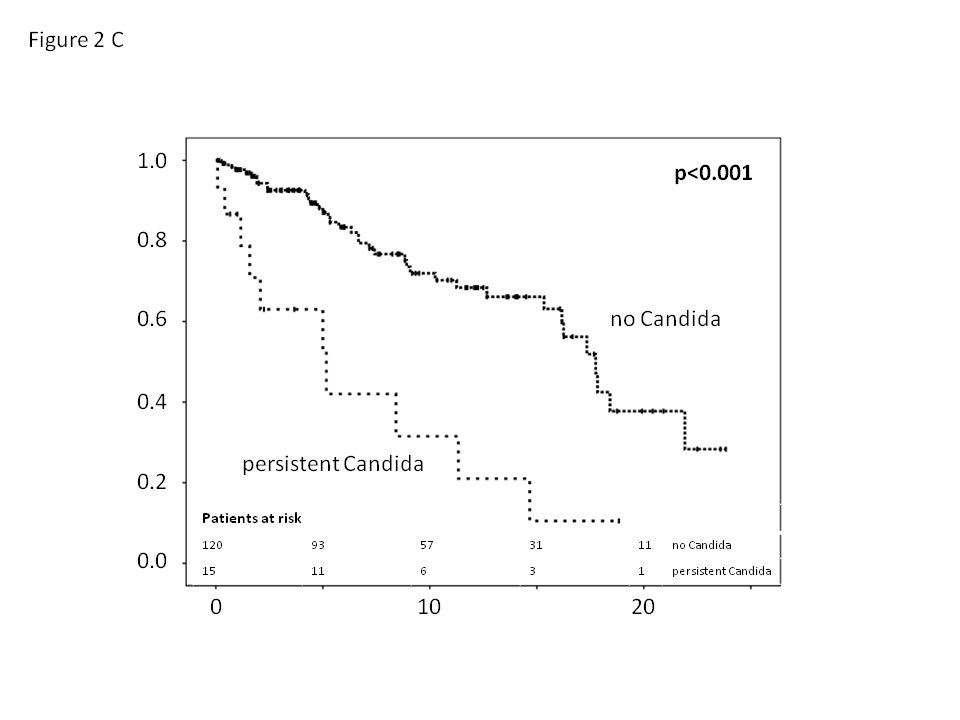

Supplement: Supplementary file 4 — Authors’ original file for figure 4 [file 12879_2014_562_MOESM4_ESM.tiff]
